# Association of Perfluoroalkyl Substances, Bone Mineral Density, and Osteoporosis in the U.S. Population in NHANES 2009–2010

**DOI:** 10.1289/ehp.1307909

**Published:** 2015-06-09

**Authors:** Naila Khalil, Aimin Chen, Miryoung Lee, Stefan A. Czerwinski, James R. Ebert, Jamie C. DeWitt, Kurunthachalam Kannan

**Affiliations:** 1 Center for Global Health, Department of Community Health, Boonshoft School of Medicine, Wright State University, Dayton, Ohio, USA; 2 Division of Epidemiology and Biostatistics, Department of Environmental Health, University of Cincinnati College of Medicine, Cincinnati, Ohio, USA; 3 Lifespan Health Research Center, Department of Community Health, Wright State University, Dayton, Ohio, USA; 4 The Pediatric Lipid Clinic, Dayton Children’s Hospital, Dayton, Ohio, USA; 5 Department of Pharmacology and Toxicology, Brody School of Medicine, East Carolina University, Greenville, North Carolina, USA; 6 Wadsworth Center, New York State Department of Health and Department of Environmental Health Sciences, University at Albany, State University of New York, Albany, New York, USA

## Abstract

**Background:**

Perfluoroalkyl substances (PFASs), including perfluorooctanoic acid (PFOA), perfluorooctane sulfonic acid (PFOS), perfluorohexane sulfonic acid (PFHxS), and perfluorononanoic acid (PFNA), are detectable in the serum of 95% of the U.S. population.

**Objective:**

Considering the role of PFASs as endocrine disruptors, we examined their relationships with bone health.

**Methods:**

The association between serum PFAS concentration and bone mineral density at total femur (TFBMD), femoral neck (FNBMD), lumbar spine (LSBMD), and physician-diagnosed osteoporosis was assessed in 1,914 participants using data from the National Health and Nutritional Examination Survey 2009–2010.

**Results:**

The mean age of the participants was 43 years. Men had higher serum PFAS concentrations than women (*p* < 0.001) except for PFNA. In both sexes, serum PFOS concentrations were inversely associated with FNBMD (*p* < 0.05). In women, significant negative associations were observed for natural log (ln)–transformed PFOS exposure with TFBMD and FNBMD, and for ln-transformed PFOA exposure with TFBMD (*p* < 0.05). In postmenopausal women, serum PFOS was negatively associated with TFBMD and FNBMD, and PFNA was negatively associated with TFBMD, FNBMD, and LSBMD (all *p* < 0.05). With one log unit increase in serum PFOA, PFHxS, and PFNA, osteoporosis prevalence in women increased as follows: [adjusted odds ratios (aORs)] 1.84 (95% CI: 1.17, 2.905), 1.64 (95% CI: 1.14, 2.38), and 1.45 (95% CI: 1.02, 2.05), respectively. In women, the prevalence of osteoporosis was significantly higher in the highest versus the lowest quartiles of PFOA, PFHxS, and PFNA, with aORs of 2.59 (95% CI: 1.01, 6.67), 13.20 (95% CI: 2.72, 64.15), and 3.23 (95% CI: 1.44, 7.21), respectively, based on 77 cases in the study sample.

**Conclusion:**

In a representative sample of the U.S. adult population, serum PFAS concentrations were associated with lower bone mineral density, which varied according to the specific PFAS and bone site assessed. Most associations were limited to women. Osteoporosis in women was also associated with PFAS exposure, based on a small number of cases.

**Citation:**

Khalil N, Chen A, Lee M, Czerwinski SA, Ebert JR, DeWitt JC, Kannan K. 2016. Association of perfluoroalkyl substances, bone mineral density, and osteoporosis in the U.S. population in NHANES 2009–2010. Environ Health Perspect 124:81–87; http://dx.doi.org/10.1289/ehp.1307909

## Introduction

Age-associated osteoporosis is a significant public health concern because it is related to bone fractures and associated morbidities (Johnell et al. 2001). It is estimated that > 9% of Americans ≥ 50 years of age had osteoporosis either at the femoral neck or at the lumbar spine in 2005–2008 [2% of all U.S. men and 10% of all U.S. women (Looker et al. 2010)]. Recent evidence suggests that exposure to environmental toxicants such as lead, cadmium, and mercury are associated with higher risks for osteoporosis and fractures (Engström et al. 2011; Khalil et al. 2008; Pollack et al. 2013).

Perfluoroalkyl substances (PFASs) have been widely used in protective water- and stain-resistant coatings on clothing, furnishings, and non-stick housewares for > 60 years. PFASs are ubiquitous environmental contaminants and are detectable in humans worldwide (Fromme et al. 2009). Of the 12 PFASs assayed in the U.S. National Health and Nutrition Examination Survey (NHANES) 1999–2008, 4 PFASs were found in 95% of the U.S. population: perfluorooctanoic acid (PFOA), perfluorooctane sulfonic acid (PFOS), perfluorohexane sulfonic acid (PFHxS), and perfluorononanoic acid (PFNA) (Calafat et al. 2007; Kato et al. 2011). The toxicity of PFASs, including tumors, liver damage, and adverse skeletal and reproductive outcomes, has been reported in animal studies (Cui et al. 2009; White et al. 2007; Yu et al. 2009). PFOA has recently been categorized as possibly carcinogenic in humans (2B classification) [International Agency for Research on Cancer (IARC) 2015]. Moreover, PFASs have been characterized as endocrine-disrupting chemicals (ED) (White et al. 2011) based on their hormonal modulation and metabolic associations (Lin et al. 2009).

A recent analysis reported negative associations between environmental exposure to PFOS and LSBMD in premenopausal women using data from NHANES 2005–2008 (Lin et al. 2014). Furthermore, experimental and human autopsy evidence suggests accumulation of PFASs in the skeleton (Bogdanska et al. 2011; Pérez et al. 2013). In the current analysis, we tested the following hypotheses: *a*) serum PFAS concentrations are negatively associated with BMD, and *b*) this association differs by sex.

## Methods

*Study methods and participants*. Publicly available data from the NHANES 2009–2010 cycle were used for this study. Detailed descriptions of the survey design and methods are available on the NHANES website [Centers for Disease Control and Prevention (CDC) 2014]. Briefly, NHANES is an ongoing survey of the non-institutionalized U.S. population collected using a stratified, multistage probability sampling design. After providing informed consent, participants visited a mobile examination center (MEC) for physical assessment, examination, and laboratory measurements. Analysis of PFASs in serum was conducted at the National Center for Environmental Health in a random, one-third subsample of participants ≥ 12 years of age. The present study sample consisted of NHANES participants 12–80 years of age who had BMD measurements available for total femur (TFBMD, *n* = 1,914), its subregion femoral neck (FNBMD, *n* = 1,914), and lumbar spine (LSBMD, *n* = 1,605), in addition to serum concentrations of four PFASs (PFOA, PFOS, PFHxS, PFNA).

*Dual X-ray absorptiometry (DXA) and osteoporosis*. BMD (grams per centimeter squared) was measured using DXA Hologic QDR 4500A fan-beam densitometers (Hologic Inc., Bedford, MA, USA) in the MEC (CDC 2014). Antero-posterior LSBMD was scanned; mean BMD was computed from the first through fourth lumbar vertebra. For TFBMD and FNBMD, the left hip was routinely scanned. If a left-hip replacement or metal objects in the left leg were reported, the right hip was scanned. Participants were excluded from the femur scan if they had bilateral hip fractures, bilateral hip replacements, or pins. Participants weighing > 300 lbs (136 kg) (DXA table limitation) and pregnant females (positive urine pregnancy test and/or self-report) were ineligible for the DXA examination. Each respondent’s scan was reviewed in the Department of Radiology, University of California, San Francisco, using standard radiologic techniques and NHANES protocols.

Participants answering “yes” to the question “Has a doctor or other health professional ever told you that you have osteoporosis?” were coded as having “self-reported physician-diagnosed” osteoporosis. The validity of self-reported osteoporosis is moderate in mid-age adults and good in older adults (Peeters et al. 2013). In a study of Australian women, the agreement between self-reported osteoporosis and medication claims was moderate in women 56–71 years of age (kappa statistic 0.51).

*PFAS assay*. Briefly, serum PFASs (non-fasting) were measured using automated solid-phase extraction coupled to isotope-dilution high–performance liquid chromatography–tandem mass spectrometry, as published elsewhere (Calafat et al. 2007). Serum measurements of four PFASs (PFOA, PFOS, PFHxS, and PFNA), which were detectable in > 98% of the 2009–2010 survey participants, were included in this analysis.

*Covariates*. Covariates selected *a priori* (Cummings et al. 1985; Hannan et al. 2000; Khalil et al. 2008) included age, race/ethnicity, sex, body mass index (BMI), smoking (serum cotinine), daily milk intake, physical activity (PA), menopause, and blood lead concentration. Sociodemographic information such as age, sex, race/ethnicity, and reproductive history were recorded using interviewer-administered questionnaires. Age was used concurrently as both a continuous variable and categorized in three levels as 12–20 years, 21–50 years, and ≥ 50 years (referent). Race/ethnicity was self-reported as non-Hispanic white, non-Hispanic black, Mexican American, other Hispanic, and other multiracial (referent).

Body weight was measured to the nearest 0.01 kg using an electronic load–cell scale, and standing height was measured with a fixed stadiometer. BMI was calculated as body weight (kilograms) divided by height (meters squared). Serum cotinine levels < 1.0 ng/mL were categorized as non-smoker (referent), 1.0–9.9 ng/mL as environmental tobacco smoke (ETS) exposure, and ≥ 10.0 ng/mL as current smoker (CDC 2015a; Hukkanen et al. 2005).

Menopause status was ascertained as self-reported cessation of regular menstruation over the past 12 months. If women responded “no” to the question “have you had regular periods in the past twelve months?” and stated that the reason for not having regular periods was due to “menopause/hysterectomy,” then they were categorized as postmenopausal.

Separate questions assessed the history of hysterectomy and bilateral oophorectomy as answering yes to a question about having had a hysterectomy [have you had a hysterectomy, including a partial hysterectomy (that is, surgery to remove your uterus or womb)?] and answering yes to a question about having had both ovaries removed (have you had both of your ovaries removed either when you had your uterus removed or at another time?), respectively. Women who answered that their reason for not having regular periods was “menopause/hysterectomy” also included women who had a hysterectomy and/or bilateral oophorectomy.

Self-reported vigorous or moderate recreational physical activity (PA) was categorized as “inactive” (< 10 consecutive minutes per week: referent), “low activity” (10 to 149 active minutes per week), “sufficient/medium activity” (150 to 299 active minutes per week, and “high activity” (≥ 300 active minutes per week) according to the 2008 Physical Activity Guidelines for Americans [Department of Health and Human Services (DHHS) 2008]. This activity categorization was derived from six PA variables using an algorithm (Tucker et al. 2011). These six variables assessed “vigorous” and “moderate” recreational PA using NHANES questionnaire data. For example, vigorous recreational PA was evaluated by participants’ answers to the following questions: *a*) “Do you do any vigorous-intensity sports, fitness, or recreational activities that cause large increases in breathing or heart rate like running or basketball for at least 10 min continuously?” *b*)“In a typical week, on how many days do you do vigorous-intensity sports, fitness or recreational activities?” *c*)“How much time do you spend doing vigorous-intensity sports, fitness or recreational activities on a typical day?” The same three variables were incorporated into the algorithm for moderate intensity PA.

Milk intake was ascertained as answering “yes” (“no”: referent) to the question, “have you been a regular (five times per week) milk drinker for most or all of your life, including childhood?” Blood lead concentration (micrograms per deciliter), and BMI were used as continuous variables [exception: see Supplemental Material, Table S1, presented as quartiles (lead), or by weight category (BMI)].

*Statistical analysis*. Population characteristics, outcomes, and exposures were summarized as the mean ± standard error (SE) or numbers of observations (percent), and differences according to sex were tested using Student’s two-tailed *t*-test or the Rao–Scott chi-square test as recommended by the National Center for Health Statistics (NCHS) (CDC 2015c). As decided *a priori*, analyses were conducted to examine the relationships between PFASs, BMD, and osteoporosis stratified by sex. Due to significant nonnormal distribution, natural log–transformation was performed on PFAS concentrations (ln-PFAS). Analyses were repeated by categorizing PFASs in quartiles using sex-specific quartile (Q1: referent, lowest; Q4: highest) cut points for men and women. We constructed full multivariable linear regression models with TFBMD, FNBMD, and LSBMD as dependent variables and individual PFAS concentrations as ln-transformed continuous predictors or in quartiles while adjusting for covariates related to BMD that have been described previously in the literature (Cauley et al. 2005, 2010). Each PFAS was modeled separately. The results are reported as regression coefficients and 95% confidence intervals (CIs).

In multiple logistic regression models, adjusted odds ratios (aORs) and 95% CIs of osteoporosis were calculated for each PFAS separately as ln-transformed predictors or as sex-specific quartiles (using the lowest quartile as referent). To explore whether PFAS, BMD, and osteoporosis associations differed by menopause status, multiple linear regression analysis stratified by menopause status was performed (as described above). In the Supplemental Material, Table S1 shows the mean untransformed PFAS concentration by covariate categories for men and women. In the Supplemental Material, Table S2 shows unadjusted mean serum PFAS concentration (ln-transformed) by osteoporosis diagnosis in men and women.

To account for the complex NHANES survey design, sampling weights, strata, and primary sampling units were adjusted in all analyses as recommended by the NCHS. SAS survey procedures were used (SAS Institute Inc., version 9.3) by applying the Taylor series linearization method to calculate SEs. Two-tailed *p*-values were used for all tests at a 5% significance level.

## Results

*Characteristics of the study population*. The mean age of the study population was 42.6 (SE: 0.6) years, with no significant difference by sex (Table 1). The study population was predominantly composed of non-Hispanic white participants. BMI was comparable by sex. A higher percentage of men were smokers and had ETS exposure (*p* < 0.001). In men, milk consumption and blood lead levels were significantly higher than in women (*p* < 0.05). Less than half of the study participants were physically inactive (42%); there was a statistically significant difference in PA between men and women (*p* < 0.001). The proportion of inactive women was higher than that of men. High PA was almost three times more common in men than in women.

**Table 1:** Characteristics of 2009–2010 NHANES study participants, distribution of serum perfluoroalkyl substances, and bone mineral density, overall and by sex.

Characteristic variable	Overall		Male		Female		*p*-Value^*a*^
	*n*	Mean ± SE or percent	*n*	Mean ± SE or percent	*n*	Mean ± SE or percent	
Age (years)	1,914	42.6 ± 0.64	956	42.0 ± 0.69	959	43.1 ± 0.68	0.139
Age groups (years)
12–20	368	15	204	15.6	164	14.5
21–50	802	50	375	50.5	427	50.4
≥ 50	744	35	377	34.0	367	35.1	0.674
BMI (kg/m^2^)	1,908	27.4 ± 0.20	953	26.8 ± 0.34	955	27.2 ± 0.24	0.277
Smoking status	1,913
Smoker	410	22	237	26.0	173	18.2
ETS	86	4.4	55	5.7	31	3.2
Nonsmokers	1,417	73.5	663	68.3	754	78.6	< 0.001^*b*^
Race/ethnicity	1,914
Non-Hispanic white	883	68	453	67.9	430	68.1
Non-Hispanic black	314	10.5	164	10.5	150	10.4
Mexican American	411	10.0	198	10.9	213	9.1
Other Hispanic	205	5.1	97	5.4	108	4.7
Other multiracial	101	6.5	44	5.3	57	7.7	0.083
Regularly drink milk 5 times/week	1,575
Yes	1,224	80	618	83	606	76
No	351	20	146	17	205	24	0.002
Recreational activity	1,905
Inactive	896	42	411	39	485	45
Low activity	290	17	120	14	170	21
Moderate activity	230	13	112	13	118	13
High activity	489	28	307	34	182	11	< 0.001
Blood lead (μg/dL)	1,914	1.43 ± 0.06	956	1.67 ± 0.08	958	1.18 ± 0.03	< 0.001
BMD total femur (g/cm^2^)	1,914	0.97 ± 0.01	956	1.03 ± 0.01	958	0.91 ± 0.01	0.001
BMD femoral neck (g/cm^2^)	1,914	0.84 ± 0.01	956	0.88 ± 0.01	958	0.81 ± 0.01	0.001
BMD lumbar spine (g/cm^2^)	1,505	1.02 ± 0.01	741	1.03 ± 0.01	764	1.01 ± 0.01	0.051
Osteoporosis	1,575
Yes	94	5	17	2	77	8
No	1,481	95	748	98	733	92	0.001
PFOA (ng/mL)^*c*^	1,914	3.7 ± 0.18	956	4.1 ± 0.21	958	3.3 ± 0.15	0.001
PFOS (ng/mL)^*c*^	1,914	12.7 ± 1.20	956	15.1 ± 1.6	958	10.3 ± 0.75	0.001
PFHxS (ng/mL)^*c*^	1,914	2.50 ± 0.10	956	3.1 ± 0.18	958	1.9 ± 0.09	0.001
PFNA (ng/mL)^*c*^	1,914	1.9 ± 0.20	956	2.0 ± 0.28	958	1.8 ± 0.13	0.134
ETS, environmental tobacco smoke. ^***a***^*p*-Values for differences between males and females: continuous variables: *t*-test; categorical variables: Rao–Scott chi-square. ^***b***^Smoking categories were based on serum cotinine concentration. ^***c***^Untransformed serum perfluoroalkyl substances.							

TFBMD and FNBMD were 12% and 8% higher in men than in women, respectively (all *p* < 0.001). LSBMD was also slightly higher in men than in women, but the difference was not statistically significant. A diagnosis of osteoporosis was reported for 5% of the total sample; however, this diagnosis was reported for only 2% of men (*n* = 17) and was reported more frequently for women (*n* = 77) (*p* < 0.001). In men, the average serum PFOA, PFOS, and PFHxS concentrations were 20%, 32%, and 36% higher than in women, respectively (*p* < 0.001 for all three). PFNA concentrations were comparable between the sexes (*p* = 0.133).

In the Supplemental Material, Table S1 summarizes covariates and PFAS concentrations by sex. A significant relationship was noted between age categories and all PFASs in both sexes except for PFHxS in men. PFOS and PFNA were significantly different across race/ethnicity in both sexes (however, this difference was only significant for PFHxS in men). PFAS exposure was not significantly related to BMI categories, recreational PA, or daily milk intake. No significant associations between smoking status and PFASs were noted except for smoking status and PFHxS in men. In women, a significant association was observed between mean blood lead levels and all PFASs. In men, only PFOS and PFHxS had a significant relationship with blood lead levels.

*Serum PFASs and BMD*. Adjusted associations between continuous ln-transformed and quartiles of PFAS and each BMD measurement are reported separately for men and women and for pre- and postmenopausal women in Tables 2–4. In most cases, ln-PFAS concentrations and BMD measurements were negatively associated in men, but only the association between ln-PFOS and FNBMD was statistically significant (β = –0.013; 95% CI: –0.024, –0.002). In general, categorical PFAS exposures were not clearly associated with any of the BMD measurements in men, except that compared with Q1, Q4 PFOS exposure had a significant negative relationship with FNBMD (β = –0.046; 95% CI: –0.078, –0.015).

**Table 2:** Multivariate*^a^* adjusted linear regression coefficients for perfluoroalkyl substances and total femur bone mineral density (TFBMD).

PFAS *n *= 1,566	Men (*n *= 956) β (95% CI)	All women (*n *= 958) β (95% CI)	Premenopausal women (*n *= 590) β (95% CI)	Postmenopausal women (*n *= 368) β (95% CI)
PFOA
Q1	Referent	Referent	Referent	Referent
Q2	–0.010 (–0.034, 0.055)	–0.020 (–0.040, –0.001)	–0.026 (–0.051, –0.001)	–0.011 (–0.059, 0.037)
Q3	–0.012 (–0.056, 0.033)	–0.002 (–0.038, 0.034)	0.006 (–0.041, 0.052)	–0.002 (–0.049, 0.045)
Q4	–0.001 (–0.042, 0.041)	–0.030 (–0.063, 0.003)	–0.029 (–0.068, 0.010)	–0.024 (–0.072, 0.024)
ln-PFOA	–0.007 (–0.028, 0.014)	–0.017 (–0.038, 0.003)	–0.017 (0.038, 0.004)	–0.012 (–0.043, 0.019)
PFOS
Q1	Referent	Referent	Referent	Referent
Q2	–0.029 (–0.074, 0.016)	–0.007 (–0.038, 0.023)	–0.013 (–0.050, 0.023)	–0.001 (–0.072, 0.069)
Q3	–0.029 (–0.063, 0.006)	–0.009 (–0.037, 0.019)	–0.017 (–0.048, 0.014)	0.002 (–0.065, 0.070)
Q4	–0.032 (–0.072, 0.008)	–0.044 (–0.074, –0.014)	–0.013 (–0.046, 0.021)	–0.059 (–0.115, –0.002)
ln-PFOS	–0.010 (–0.027, 0.006)	–0.018 (–0.034, –0.002)	–0.004 (–0.020, 0.012)	–0.033 (–0.049, –0.015)
PFHxS
Q1	Referent	Referent	Referent	Referent
Q2	–0.004 (–0.046, 0.038)	–0.007 (–0.038, 0.023)	–0.013 (–0.050, 0.023)	–0.001 (–0.072, 0.069)
Q3	–0.004 (–0.043, 0.036)	–0.009 (–0.037, 0.019)	–0.017 (–0.048, 0.014)	0.002 (–0.065, 0.070)
Q4	–0.026 (–0.065, 0.013)	–0.044 (–0.074, –0.014)	–0.013 (–0.046, 0.021)	–0.059 (–0.115, –0.002)
ln-PFHxS	–0.010 (–0.025, 0.004)	–0.012 (–0.026, 0.002)	–0.009 (–0.026, 0.007)	–0.009 (–0.029, 0.011)
PFNA
Q1	Referent	Referent	Referent	Referent
Q2	–0.003 (–0.053, 0.047)	–0.017 (–0.050, 0.016)	–0.017 (–0.054, 0.020)	–0.008 (–0.070, 0.053)
Q3	–0.006 (–0.039, 0.026)	–0.008 (–0.041, 0.026)	–0.004 (–0.046, 0.038)	0.001 (–0.060, 0.060)
Q4	0.007 (–0.031, 0.045)	–0.040 (–0.077, –0.003)	–0.039 (–0.071, –0.006)	–0.023 (–0.083, 0.042)
ln-PFNA	–0.006 (–0.030, 0.018)	–0.017 (–0.038, 0.003)	–0.009 (–0.028, 0.010)	–0.027 (–0.053, –0.002)
^***a***^Adjusted for age (continuous) and age categories (12–20, 21–50, ≥ 50 years), ethnicity, BMI, serum cotinine, physical activity, milk consumption, and blood lead concentration.				

**Table 3:** Multivariate*^a^* adjusted linear regression coefficients for perfluoroalkyl substances and total femur neck mineral density (FNBMD).

PFAS *n *= 1,566	Men (*n *= 956) β (95% CI)	All women (*n *= 958) β (95% CI)	Premenopausal women (*n *= 590) β (95% CI)	Postmenopausal women (*n *= 368) β (95% CI)
PFOA
Q1	Referent	Referent	Referent	Referent
Q2	0.011 (–0.021, 0.043)	–0.025 (–0.052, 0.002)	–0.028 (–0.060, 0.003)	–0.022 (–0.077, 0.033)
Q3	–0.013 (–0.053, 0.028)	–0.002 (–0.039, 0.034)	0.014 (–0.031, 0.060)	–0.024 (–0.083, 0.035)
Q4	0.004 (–0.035, 0.043)	–0.028 (–0.058, 0.001)	–0.019 (–0.056, 0.018)	–0.041 (–0.098, 0.016)
ln-PFOA	0.001 (–0.025, 0.022)	–0.017 (–0.033, –0.001)	–0.012 (–0.030, 0.007)	0.020 (–0.049, 0.010)
PFOS
Q1	Referent	Referent	Referent	Referent
Q2	–0.036 (–0.077, 0.006)	0.001(–0.019, 0.019)	–0.005 (–0.028, 0.018)	–0.005 (0.087, 0.077)
Q3	–0.027 (–0.063, 0.009)	–0.001 (–0.025, 0.025)	–0.005 (–0.028, 0.017)	–0.001 (–0.082, 0.080)
Q4	–0.046 (–0.078, –0.015)	–0.034 (–0.059, –0.009)	–0.001 (–0.029, 0.029)	–0.062 (0.134, 0.009)
ln-PFOS	–0.013 (–0.024, –0.002)	–0.016(–0.029, –0.002)	–0.001 (–0.015, 0.015)	–0.033 (–0.049, –0.017)
PFHxS
Q1	Referent	Referent	Referent	Referent
Q2	–0.002 (–0.042, 0.038)	–0.008 (–0.039, 0.023)	–0.004 (–0.042, 0.033)	–0.025 (–0.100, 0.050)
Q3	–0.004 (–0.031, 0.023)	0.001 (–0.025, 0.027)	0.010 (–0.018, 0.038)	–0.017 (–0.092, 0.058)
Q4	–0.013 (–0.052, 0.025)	–0.018 (–0.051, 0.016)	–0.010 (–0.039, 0.018)	–0.026 (–0.104, 0.051)
ln-PFHxS	–0.009 (–0.024, 0.006)	–0.005 (–0.018, 0.008)	–0.001 (–0.015, 0.013)	–0.005 (–0.024, 0.013)
PFNA
Q1	Referent	Referent	Referent	Referent
Q2	–0.010 (–0.058, 0.037)	–0.020 (–0.066, 0.027)	–0.023 (–0.073, 0.027)	–0.013 (–0.087, 0.060)
Q3	–0.004 (–0.035, 0.028)	–0.001 (–0.029, 0.029)	0.001 (–0.035, 0.038)	–0.009 (–0.078, 0.060)
Q4	0.009 (–0.022, 0.039)	–0.023 (–0.051, 0.005)	–0.005 (–0.049, 0.040)	–0.046 (–0.103, 0.012)
ln-PFNA	–0.005 (–0.021, 0.018)	–0.014 (–0.032, 0.003)	–0.005 (–0.025, 0.016)	–0.025 (–0.049, –0.001)
^***a***^Adjusted for age (continuous) and age categories (12–20, 21–50, ≥ 50 years), ethnicity, BMI, serum cotinine, physical activity, milk consumption, and blood lead concentration.				

**Table 4:** Multivariate*^a^* adjusted linear regression coefficients for perfluoroalkyl substances and lumbar spine bone mineral density (LSBMD).

PFAS *n *= 1,566	Men (*n *= 956) β (95% CI)	All women (*n *= 958) β (95% CI)	Premenopausal women (*n *= 590) β (95% CI)	Postmenopausal women (*n *= 368) β (95% CI)
PFOA
Q1	Referent	Referent	Referent	Referent
Q2	0.013 (–0.042, 0.068)	–0.008 (–0.035, 0.019)	–0.008 (–0.041, 0.025)	–0.001 (–0.089, 0.088)
Q3	–0.023 (–0.083, 0.037)	0.015 (–0.019, 0.049)	0.020 (–0.020, 0.060)	0.011 (–0.090, 0.113)
Q4	–0.005 (–0.058, 0.049)	–0.020 (–0.049, 0.009)	–0.010 (–0.042, 0.021)	–0.017 (–0.111, 0.077)
ln-PFOA	–0.011 (–0.039, 0.017)	–0.009 (–0.029, 0.011)	0.001 (–0.020, 0.021)	–0.017 (–0.058, 0.024)
PFOS
Q1	Referent	Referent	Referent	Referent
Q2	–0.023 (–0.064, 0.018)	0.001 (–0.038, 0.040)	0.001 (–0.044, 0.045)	–0.040 (–0.165, 0.085)
Q3	–0.026 (–0.066, 0.014)	0.008 (–0.024, 0.039)	0.009 (–0.026, 0.045)	–0.023 (–0.144, 0.097)
Q4	–0.023 (–0.064, 0.017)	–0.011 (–0.053, 0.032)	0.015 (–0.022, 0.052)	0.058 (–0.192, 0.075)
ln-PFOS	–0.011 (–0.028, 0.006)	–0.003 (–0.022, 0.017)	0.010 (–0.008, 0.027)	–0.019 (–0.047, 0.009)
PFHxS
Q1	Referent	Referent	Referent	Referent
Q2	0.015 (–0.021, 0.050)	0.017 (–0.019, 0.053)	0.026 (–0.017, 0.069)	–0.017 (–0.103, 0.069)
Q3	0.021 (–0.015, 0.057)	0.026 (–0.013, 0.065)	0.028 (–0.017, 0.073)	0.035 (–0.067, 0.137)
Q4	0.005 (–0.022, 0.033)	–0.015 (–0.046, 0.016)	–0.014 (–0.043, 0.015)	0.001 (–0.089, 0.091)
ln-PFHxS	0.001 (–0.011, 0.012)	–0.003 (–0.015, 0.009)	0.003 (–0.013, 0.019)	–0.001 (–0.021, 0.020)
PFNA
Q1	Referent	Referent	Referent	Referent
Q2	0.004 (–0.046, 0.054)	–0.013 (–0.078, 0.052)	–0.009 (–0.069, 0.050)	–0.016 (–0.145, 0.113)
Q3	–0.013 (–0.056, 0.029)	0.005 (–0.034, 0.043)	0.009 (–0.021, 0.039)	–0.022 (–0.134, 0.098)
Q4	0.009 (–0.026, 0.044)	–0.023 (–0.057, 0.012)	0.004 (–0.035, 0.043)	–0.061 (–0.170, 0.048)
ln-PFNA	–0.006 (–0.029, 0.017)	–0.016 (–0.032, 0.001)	–0.016 (–0.032, 0.001)	–0.043 (–0.073, –0.013)
^***a***^Adjusted for age (continuous) and age categories (12–20, 21–50, ≥ 50 years), ethnicity, BMI, serum cotinine, physical activity, milk consumption, and blood lead concentration.				

In women, ln-PFOS was associated with significantly lower TFBMD and FNBMD, and ln-PFOA was associated with significantly lower TFBMD. In premenopausal women, no significant association was observed between any PFASs and BMD. In postmenopausal women, serum ln-PFOS was significantly associated with TFBMD and FNBMD; PFNA was inversely related with TFBMD, FNBMD, and LSBMD (all *p* < 0.05).

In quartile analyses, women showed significant negative associations between TFBMD and Q2 of PFOA, Q4 of PFOS, and Q4 of PFHxS; and between FNBMD and Q4 of PFOS, relative to the lowest quartile (Q1) of each exposure. In premenopausal women, TFBMD also had significant negative associations with Q2 of PFOA and Q4 of PFNA. In postmenopausal women, TFBMD was significantly lower in Q4 of PFOS and PFHxS. LSBMD was not significantly associated with any of the categorical PFAS exposures in men or in women, regardless of menopausal status.

*Serum PFAS and osteoporosis*. There were 17 cases of osteoporosis in men (Table 1), and there were no significant differences in mean PFAS levels according to case status in men (see Supplemental Material, Table S2). In women, the mean values of all four PFASs were significantly higher in the 77 women with osteoporosis than in the 733 women who did not report osteoporosis. The aORs for PFOA and PFNA were significant for both the continuous ln-transformed exposures (aOR = 1.84; 95% CI: 1.17, 2.90 and aOR = 1.45; 95% CI: 1.02, 2.05, respectively) and for the Q4 versus Q1 comparisons (aOR = 2.59; 95% CI: 1.01, 6.67 and aOR = 3.23; 95% CI: 1.44, 7.21, respectively) (Table 5). In addition, ln-PFHxS was significantly associated with osteoporosis in both the ln-PFAS model (aOR 1.64; 95% CI: 1.14, 2.38) and the quartile analysis, with significant associations for all three quartiles (e.g., aOR = 13.20; 95% CI: 2.72, 64.15 for Q4 vs. Q1). However, there were only eight women with osteoporosis in Q2 of PFHxS, and quartile-specific aORs were very imprecise. PFOS was not significantly associated with osteoporosis in the continuous or quartile exposure models.

**Table 5:** Multivariate*^a^* logistic regression of perfluoroalkyl substances and osteoporosis in women.

PFAS	Osteoporosis (*n*)	No osteoporosis (*n*)	Odds ratio (95% CI)	*p*-Value^*b*^
PFOA
Q1	8	175	Referent	—
Q2	16	186	1.25 (0.38, 4.06)	0.713
Q3	17	178	1.23 (0.37, 4.05)	0.734
Q4	36	194	2.59 (1.01, 6.67)	0.049
ln-PFOA	77	733	1.84 (1.17, 2.90)	0.008
PFOS
Q1	11	175	Referent	—
Q2	8	186	0.42 (0.13, 1.32)	0.137
Q3	22	190	0.83 (0.45, 1.51)	0.540
Q4	36	184	1.07 (0.36, 3.19)	0.908
ln-PFOS	77	733	1.14 (0.68, 1.94)	0.619
PFHxS
Q1	11	175	Referent	—
Q2	8	184	9.29 (1.81, 47.62)	0.008
Q3	22	190	8.06 (1.84, 35.25)	0.006
Q4	36	184	13.20 (2.72, 64.15)	0.001
ln-PFHxS	77	733	1.64 (1.14, 2.38)	0.008
PFNA
Q1	11	175	Referent	—
Q2	8	184	1.93 (0.72, 5.10)	0.191
Q3	22	190	0.82 (0.25, 2.64)	0.735
Q4	36	184	3.23 (1.44, 7.21)	0.004
ln-PFNA	77	733	1.45 (1.02, 2.05)	0.001
^***a***^Adjusted for age (continuous) and age categories (12–20, 21–50, ≥ 50 years), ethnicity, BMI, serum cotinine, physical activity, milk consumption, and blood lead concentration. ^***b***^Wald Chi-square *p*-values.				

## Discussion

In this representative sample of the U.S. population, PFOS and PFHxS were associated with lower TFBMD and a higher prevalence of osteoporosis among women. In addition, PFOS was negatively associated with FNBMD, PFOA was negatively associated with TFBMD, and PFNA was positively associated with osteoporosis only. In general, associations were stronger among postmenopausal women than among premenopausal women. In men, PFOS was associated with lower FNBMD only. LSBMD was not clearly associated with any of the PFASs in men or women. Although the variance in BMD explained by PFAS was very small (*R*^2^ < 1%) in this exploratory investigation, given the study sample size and the nationally representative data, these results are important and support the need for further research to evaluate PFAS toxicity with regard to bone health.

Human exposure to PFASs has gradually increased since the 1950s (Cousins 2013), although following regulatory efforts, serum concentrations of some PFASs have decreased in recent years (Kato et al. 2011). PFASs are present in many food items including meat, poultry, eggs, fish, and fresh produce, and diet contributes significantly to daily human exposure to PFASs (Domingo 2012). PFASs are poorly metabolized and slowly eliminated from the human body, with half-lives of 4–8 years (Kato et al. 2011), and they partition to bone tissue (Pérez et al. 2013; Bogdanska et al. 2011).

Limited data from animal models suggest PFAS toxicity to bone. Prenatal PFOS exposure in rodents was associated with fetal bone malformation [Thibodeaux et al. 2003; Organisation for Economic Co-operation and Development (OECD) 2002]. In mice, environmentally relevant doses of PFOS were rapidly deposited in bone (Bogdanska et al. 2011). Recent data from human autopsy studies suggest that PFASs are sequestered in bone, with PFOA being predominant (Pérez et al. 2013). Taken together, these studies suggest that PFASs are deposited in bone and may induce osteotoxicity.

PFASs at low doses are categorized as endocrine-disrupting chemicals (EDCs) in animals, including rats (Shi Z et al. 2009a, 2009b;), mice (Zhao et al. 2010), and fish (Shi X et al. 2009), and have been characterized as such in some human studies (Knox et al. 2011b; Louis et al. 2012; White et al. 2011). Data from animal models suggest that bone tissue could be an important target for a number of EDC environmental pollutants (Agas et al. 2013; Finnilä et al. 2010; Kamei et al. 2008) because EDCs can disturb the complex hormonal control of bone metabolism. For example, sex-dependent associations of another EDC, polychlorinated biphenyl (PCB), with bone length have been reported in female sheep fetuses (Gutleb et al. 2010). In another study, pregnant ewes exposed to multiple EDCs showed reduced BMD (Lind et al. 2010).

Epidemiological research supports the EDC hypothesis for some forms of PFAS toxicity. PFAS exposure was related to delayed onset of puberty (Lopez-Espinosa et al. 2011). Older age at menarche has been associated with an increased risk of fractures in women of approximately 20 years of age (Chevalley et al. 2012), with reduced BMD in perimenopausal women (Tuppurainen et al. 1995), and with an increased risk of hip fractures in older women (Paganini-Hill et al. 2005). Moreover, serum PFOA and PFOS (specifically) were associated with earlier age at menopause in the C8 Health Project (Knox et al. 2011b) and in the NHANES (Taylor et al. 2014). Knox et al. (2011b) reported that serum PFOS was negatively associated with serum estradiol concentration. Another pathway that may link PFASs and BMD is through thyroid hormone modulation. Thyroid hormones play a crucial role in bone health and remodeling (Lee et al. 2010). PFAS exposure was associated with serum thyroxine (T_4_) and triiodothyronine (T_3_) levels in two cross-sectional studies (Knox et al. 2011a; Wen et al. 2013) and with altered responses to T_3_ in a T_3_-dependent cell line *in vitro* (Long et al. 2013). Based on a cross-sectional study of adult NHANES participants, Wen et al. (2013) reported that serum PFHxS was positively associated with subclinical hyperthyroidism (defined as TSH < 0.24 mIU/mL) in women, which is a risk factor for osteoporosis (El Hadidy et al. 2011).

In the present analysis, men had higher serum levels of PFASs, but women experienced a greater BMD deficit and osteoporosis risk than did men. These observations corroborate the recently published findings of Lin et al. (2014), who reported that PFOS exposure was related to a BMD deficit only in women. The difference between the sexes with regard to the association between PFASs and BMD suggests that reproductive hormones may be involved. Females may be more sensitive to PFAS toxicity than males, or, as animal studies suggest, PFASs may be eliminated differently by males and females (Betts 2007).

We observed a higher prevalence of osteoporosis associated with PFOA, PFNA, and PFHxS in women. The odds ratios and 95% CIs of osteoporosis were large but imprecise for PFHxS in the quartile analysis owing to the small number of observations. We are unable to explain this strong association, and because of the limited precision of the estimates, the associations should be interpreted with caution. PFHxS has the longest half-life (8.5 years) of the four PFASs and has recently been associated with impaired thyroid function and early menopause in epidemiological studies (Taylor et al. 2014; Wen et al. 2013).

One of the study limitations was its cross-sectional design; we cannot confirm that the exposures preceded the outcomes of interest or rule out the possibility of reverse causation (Taylor et al. 2014). The strengths of this study include a large sample representative of the U.S. population. Four PFASs commonly detected in U.S. residents were assayed, and to our knowledge, this is the first report assessing the relationship of four PFASs with BMD at three bone sites; the only such previous study, by Lin et al. (2014), was limited to two PFASs (PFOA, PFOS) and BMD at the lumbar spine and total hip.

We observed significant negative associations between PFOS, PFNA, and PFHxS and TFBMD, FNBMD, and osteoporosis in women. Lin et al. (2014) reported a significant negative association between PFOS and LSBMD in premenopausal women only; however, we did not observe any significant association between PFOS and LSBMD in our sample of premenopausal women.

Some potential reasons for the discrepant relationships between PFOS and LSBMD observed in the present study and those observed by Lin et al. (2014) could be attributed to the differences in the NHANES survey cycles examined and to differences in sample size, age range, and covariates included. For example, the study by Lin et al. (2014) used data from two combined NHANES surveys, 2005–2006 and 2007–2008, whereas our sample consisted of data obtained from one NHANES survey conducted in 2009–2010. Furthermore, the decreasing mean serum PFOS concentration in the U.S. population could have masked an association with LSBMD in our study. As shown in the NHANES surveys, geometric mean (GM) serum PFOS levels in the general U.S. population, including women, decreased over the 2005–2006 (overall: 17.1 ng/mL, women: 14.4 ng/mL), 2007–2008 (overall: 13.2 ng/mL, women: 10.7 ng/mL), and 2009–2010 (overall: 9.3 ng/mL, women: 7.7 ng/mL) (CDC 2015b) surveys.

## Conclusion

In conclusion, our findings indicate that some PFASs are associated with low BMD and an increased prevalence of osteoporosis in U.S. women. However, these findings must be interpreted with caution given the cross-sectional study design, the large number of comparisons made, and the small number of osteoporosis cases in the study population.

## Supplemental Material

(254 KB) PDF
