# Supplementary material for: Association of Perfluoroalkyl Substances, Bone Mineral Density, and Osteoporosis in the U.S. Population in NHANES 2009–2010
Source: Environ Health Perspect. 2015 Jun 9;124(1):81–7. doi: 10.1289/ehp.1307909 (PMC4710590; doi:10.1289/ehp.1307909)
Supplement: (254 KB) PDF [file ehp.1307909.s001.acco.pdf]

Environ Health Perspect
DOI: 10.1289/ehp.1307909

Note to Readers: *EHP* strives to ensure that all journal content is accessible to all readers. However, some figures and Supplemental Material published in *EHP* articles may not conform to 508 standards due to the complexity of the information being presented. If you need assistance accessing journal content, please contact ehp508@niehs.nih.gov. Our staff will work with you to assess and meet your accessibility needs within 3 working days.

Supplemental Material

Association of Perfluoroalkyl Substances, Bone Mineral Density, and Osteoporosis in the U.S. Population in NHANES 2009–2010

Naila Khalil, Aimin Chen, Miryoung Lee, Stefan A. Czerwinski, James R. Ebert, Jamie C. DeWitt, and Kurunthachalam Kannan

Table of Contents

Table S1. Mean serum concentrations of perfluoroalkyl substances (± SE) in men and women.

Table S2. Unadjusted Mean Serum Concentrations of Perfluoroalkyl Substances by Osteoporosis Diagnosis in Men and Women (log transformed PFASs).

Table S1. Mean serum concentrations of perfluoroalkyl substances (± SE) in men and women.

	**PFOA**		**PFOS**		**PFHxS**		**PFNA**	
Characteristic	Male	Female	Male	Female	Male	Female	Male	Female
**Age (years)**								
12-20	3.20 ± 0.14	2.80 ± 0.17	9.03±0.77	7.31±0.49	3.75±0.31	2.71±0.35	1.63±0.16	1.39±0.08
21-50	4.17 ± 0.24	2.98 ± 0.17	14.00±1.65	8.35±0.96	3.09±0.25	1.29±0.08	2.04±0.25	1.67±0.15
>50	4.29 ± 0.28	3.88 ± 0.19	19.48±2.24	14.38±0.87	2.74±0.19	2.59±0.15	2.21±0.38	2.09±0.18
P-value	<0.001	<0.001	<0.001	<0.001	0.118	<0.001	0.064	<0.001
**Race/ethnicity**								
Non-Hispanic White	4.34 ± 0.28	3.51 ± 0.20	15.54 ± 2.06	10.62 ± 1.20	3.39 ± 0.22	2.22 ± 0.11	2.07± 0.36	1.77± 0.18
Non-Hispanic Black	3.89 ± 0.28	2.94 ± 0.15	16.41 ± 1.37	11.20 ± 1.42	3.03 ± 0.37	1.56 ± 0.11	2.17 ± 0.15	2.05 ± 0.16
Mexican American	3.22 ± 0.15	2.23 ± 0.15	10.47 ± 0.71	6.51 ± 0.52	1.97 ± 0.15	1.25 ± 0.25	1.64 ± 0.06	1.25 ± 0.07
Other Hispanic	3.20 ± 0.13	2.59 ± 0.32	8.63 ± 0.54	6.85 ± 0.53	1.73 ± 0.16	1.30 ± 0.09	1.78 ± 0.16	1.71 ± 0.19
Other Multiracial	3.33 ± 0.28	3.16 ± 0.57	22.74 ± 6.40	13.06 ± 1.61	2.66 ± 0.37	1.33 ± 0.22	2.35 ± 0.41	2.10 ± 0.25
P-value	0.067	0.117	0.006	0.002	0.012	0.754	0.029	<0.001
**BMI(Kg/m^2)**								
Underweight	3.55 ± 0.54	3.70 ± 0.45	13.22 ± 1.87	11.06 ± 2.44	4.20 ± 0.79	2.42 ± 0.49	1.81 ± 0.21	1.44 ± 0.14
Normal weight	4.09 ± 0.19	3.11 ± 0.17	15.23 ± 1.46	9.62 ± 0.67	3.32 ± 0.24	2.01 ± 0.16	1.89 ± 0.15	1.67 ± 0.10
Overweight	4.09 ± 0.22	3.45 ± 0.19	15.07 ± 1.89	10.17 ± 0.75	2.77 ± 0.17	1.87 ± 0.11	2.05 ± 0.32	1.87 ± 0.18
Obese	4.04 ± 0.31	3.34 ± 0.21	15.17 ± 2.16	11.23 ± 1.35	3.10 ± 0.37	1.89 ± 0.17	2.17 ± 0.38	1.88 ± 0.22
P-value	0.863	0.543	0.866	0.308	0.347	0.481	0.286	0.102
**Smoking status** [a]								
Smoker	4.31 ± 0.25	3.42 ± 0.25	16.05 ± 2.01	9.39 ± 0.78	3.42 ± 0.24	1.80 ± 0.13	2.17 ± 0.32	1.69 ± 0.14
ETS	3.68 ± 0.22	3.18 ± 0.43	11.38 ± 1.13	9.90 ± 1.62	3.91 ± 0.55	1.97 ± 0.40	1.66 ± 0.11	1.61 ± 0.20
Non-smoker	3.99 ± 0.23	3.23 ± 0.15	15.03 ± 1.71	10.55 ± 0.88	2.87 ± 0.21	1.99 ± 0.11	2.01 ± 0.28	1.80 ± 0.14
P-value	0.138	0.389	0.588	0.265	0.041	0.374	0.079	0.216
**Regularly drink milk 5 times per week**								
No	4.26 ± 0.34	3.51± 0.25	14.54 ± 2.13	11.48 ± 1.52	2.95 ± 0.52	1.84 ± 0.15	2.24 ± 0.39	1.94 ± 0.25
Yes	4.18 ± 0.24	3.29 ± 0.14	16.37 ± 1.77	10.42 ± 0.18	2.94 ± 0.34	1.83 ± 0.09	2.07 ± 0.29	1.80 ± 0.13
P-value	0.745	0.201	0.112	0.423	0.989	0.944	0.114	0.403
**Recreational activity**								
Inactive	4.31 ± 0.29	3.21 ±0.26	18.45 ± 2.87	10.61 ± 1.87	3.26 ± 0.42	2.08 ± 0.23	2.24 ± 0.36	1.83 ±0.30
Low activity	3.97 ± 0.19	3.40 ± 0.30	13.33 ± 0.66	11.17 ± 1.65	2.72 ± 0.41	1.91 ± 0.34	1.92 ± 0.17	1.61 ± 0.21
Moderate activity	4.12 ± 0.20	3.28 ± 0.12	13.97 ± 1.43	8.63 ± 0.54	3.41 ± 0.21	2.12 ± 0.24	1.98 ± 0.23	1.63 ± 0.06
Hi activity	3.98 ± 0.31	3.25 ± 0.19	15.51 ± 2.07	10.61 ± 0.67	2.81 ± 0.21	1.83 ± 0.21	2.05 ± 0.34	1.86 ± 0.13
P-value	0.409	0.968	0.461	0.793	0.425	0.248	0.470	0.723
**Blood lead (ug/dL) quartiles**								
<0.75	3.69 ± 0.18	2.87 ± 0.13	11.17 ± 0.71	7.94±0.62	3.67± 0.43	1.92 ± 0.15	1.72 ±0.12	1.42±0.07
0.75- <1.14	3.94 ± 0.23	3.00 ± 0.21	13.22 ± 1.11	9.75±1.04	3.19± 0.30	1.64 ± 0.12	1.73 ± 0.14	1.73±0.17
1.14-<1.88	4.30 ± 0.33	3.76 ± 0.26	16.06 ± 2.56	12.01± 0.81	3.84± 0.25	2.01± 0.14	2.26±0.45	1.99±0.15
>=1.88	4.16 ± 0.29	3.91 ± 0.34	18.28 ± 1.90	14.03±1.97	2.82± 0.14	2.45± 0.13	2.29±0.35	2.33±0.37
p-value	0.150	0.001	<0.001	<0.001	0.017	0.021	0.058	0.001

[a]Smoking categories based on serum cotinine concentration, ETS:environmental tobacco smoke.

Table S2. Unadjusted Mean Serum Concentrations of Perfluoroalkyl Substances by Osteoporosis Diagnosis in Men and Women (log transformed PFASs).

	Men			Women		
**Mean ± SE**	**Osteoporosis, Yes n=17**	**Osteoporosis, No n=748**	**p-value [a]**	**Osteoporosis, Yes n=77**	**Osteoporosis, No n=733**	**p-value [a]**
**PFOA**	1.34 ± 0.16	1.29 ± 0.05	0.743	1.34 ± 0.07	0.98 ± 0.05	<0.001
**PFOS**	2.37 ± 0.16	2.51 ± 0.07	0.452	2.47 ± 0.13	2.03 ± 0.06	0.007
**PFHxS**	0.70 ± 0.27	0.79 ± 0.06	0.731	0.78 ± 0.10	0.17 ± 0.06	<0.001
**PFNA**	0.28 ± 0.10	0.53 ± 0.08	0.066	0.57 ± 0.11	0.36 ± 0.05	0.034

[a] t-test adjusted for survey weights
